# The reductase domain in a Type I fatty acid synthase from the apicomplexan *Cryptosporidium parvum*: Restricted substrate preference towards very long chain fatty acyl thioesters

**DOI:** 10.1186/1471-2091-11-46

**Published:** 2010-11-22

**Authors:** Guan Zhu, Xiangyu Shi, Xiaomin Cai

**Affiliations:** 1 Department of Veterinary Pathobiology, College of Veterinary Medicine & Biomedical Sciences, Texas A&M University, College Station, Texas 77843-4467, USA; 2 Faculty of Genetics Program, Texas A&M University, College Station, Texas 77843-4467, USA

## Abstract

**Background:**

The apicomplexan *Cryptosporidium parvum *genome possesses a 25-kb intronless open reading frame (ORF) that predicts a multifunctional Type I fatty acid synthase (CpFAS1) with at least 21 enzymatic domains. Although the architecture of CpFAS1 resembles those of bacterial polyketide synthases (PKSs), this megasynthase is predicted to function as a fatty acyl elongase as our earlier studies have indicated that the N-terminal loading unit (acyl-[ACP] ligase) prefers using intermediate to long chain fatty acids as substrates, and each of the three internal elongation modules contains a complete set of enzymes to produce a saturated fatty acyl chain. Although the activities of almost all domains were confirmed using recombinant proteins, that of the C-terminal reductase domain (CpFAS1-R) was yet undetermined. In fact, there were no published studies to report the kinetic features of any reductase domains in bacterial PKSs using purified recombinant or native proteins.

**Results:**

In the present study, the identity of CpFAS1-R as a reductase is confirmed by in silico analysis on sequence similarity and characteristic motifs. Phylogenetic analysis based on the R-domains supports a previous notion on the bacterial origin of apicomplexan Type I FAS/PKS genes. We also developed a novel assay using fatty acyl-CoAs as substrates, and determined that CpFAS1-R could only utilize very long chain fatty acyl-CoAs as substrates (i.e., with activity on C26 > C24 > C22 > C20, but no activity on C18 and C16). It was capable of using both NADPH and NADH as electron donors, but prefers NADPH to NADH. The activity of CpFAS1-R displayed allosteric kinetics towards C26 hexacosanoyl CoA as a substrate (***h ***= 2.0; ***V***_max _= 32.8 nmol min^-1 ^mg^-1 ^protein; and ***K***_50 _= 0.91 mM).

**Conclusions:**

We have confirmed the activity of CpFAS1-R by directly assaying its substrate preference and kinetic parameters, which is for the first time for a Type I FAS, PKS or non-ribosomal peptide synthase (NRPS) reductase domain. The restricted substrate preference towards very long chain fatty acyl thioesters may be an important feature for this megasynthase to avoid the release of product(s) with undesired lengths.

## Background

*Cryptosporidium *is a group of important parasites that infect a wide range of hosts from reptiles and birds to humans and other mammals [1-3]. Among them, *C. parvum *is zoonotic and infects both humans and animals. *Cryptosporidium *infection in immunocompetent individuals may cause self-limiting diarrhea, but its infection in immunocompromized patients can be chronic and life-threatening [4,5]. Therefore, it is an important opportunistic pathogens in AIDS patients. Additionally, there are no effective treatments against cryptosporidial infection in AIDS patients.

**Figure 1:**
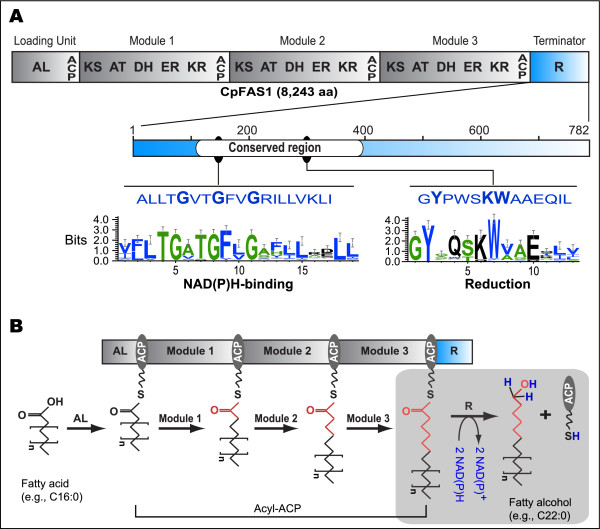
**Structure of the reductase (**R) **domain within CpFAS1 and its catalyzed reaction**. **A**) Illustration of the position and conserved NAD(P)H-binding and reductive motifs of the **R **domain within the CpFAS1 megasynthase. **B) **Proposed reactions catalyzed by CpFAS1 including that by the **R **domain. Newly elongated carbon chains are shown in red, while the hydrogen groups donated from NAD(P)H are shown in blue.

The *Cryptosporidium *genus belongs to the Phylum Apicomplexa that also contains many important human and animal parasites such as *Plasmodium, Theileria, Toxoplasma, Eimeria *and *Cyclospora *[6]. However, *Cryptosporidium *is known to be evolutionarily and metabolically divergent from other apicomplexans. For example, as a group of early branch apicomplexans, *Cryptosporidium *lacks an apicoplast and its associated metabolic pathways (e.g., isoprenoid and Type II fatty acid synthetic pathways). It has also virtually lost the capacity to synthesize any nutrients de novo [6-9]. On the other hand, this parasite possesses a unique Type I fatty acid synthase (CpFAS1) and a putative polyketide synthase (CpPKS1) that are encoded by 25-kb and 40-kb intronless open reading frames (ORFs), respectively [10-12]. The megasynthase CpFAS1 is defined by at least 21 enzymatic domains including a loading unit (containing a fatty acyl ligase [AL] and an acyl carrier protein [ACP]); three internal module, each containing a ketoacyl synthase [KS], an acyltransferase [AT], a dehydrase [DH], an enoyl reductase [ER], a ketoacyl reductase [KR] and an ACP; and a C-terminal acyl reductase domain (**R**) (Figure 1A) [10]. The AL domain loads a fatty acid to the ACP to form an acyl-ACP, in which the acyl chain may be elongated by the internal modules and finally released by the **R **domain (Figure 1B).

Due to the extreme large size of CpFAS1, it is impractical to express the entire megasynthase for biochemical analysis. Therefore, we have adapted a "divide and conquer" strategy and successfully expressed individual units/modules to study their biochemical features [13]. Our previous experiments using recombinant proteins have shown that the CpFAS1 loading unit has a substrate preference to medium and long chain fatty acids (MCFAs and LCFAs), and all domains within the three internal modules are enzymatically functional, suggesting that CpFAS1 is likely involved in the elongation of saturated fatty acid(s), rather than the de novo fatty acid synthesis [12,13]. However, although the C-terminal **R **domain was expressed, its biochemical features were yet to be determined then due to the lack of an appropriate assay.

Here we report our recently developed assay and experiments that reveal some unique biochemical features for the CpFAS1 acyl reductase domain (CpFAS1-R) including the substrate preference and kinetics for the first time for this family of acyl reductases. We have not only confirmed the reductive activity of CpFAS1-R, but also determined that it could only utilize very long chain fatty acyl thioesters as its substrates. Additionally, we have also performed phylogenetic analysis and observed bacterial-affinity of reductase domains from various apicomplexan FASs and PKSs.

## Results

### Sequence features and evolutionary affiliation of the CpFAS1-R domain

Among apicomplexans, Type I FAS/PKS genes are only discovered from *Cryptosporidium *and cyst-forming and intestinal coccidia (eg. *Toxoplasma *and *Eimeria*), but absent in the hematozoa that contain only Type II FAS (eg. *Plasmodium *and *Babesia*) or no FAS at all (eg. *Theileria*) [7,12,14-16]. Both *Cryptosporidium *and *Toxoplasma *genomes contain reductase domains at the C-terminal ends of the Type I FAS and PKS, respectively. The *E. tenella *genome is only partially sequenced (see http://www.sanger.ac.uk/Projects/E_tenella/), from which only one reductase domain could be recovered. The architectures of CpFAS1 and CpPKS1 resemble bacterial and fungal PKS rather than the Type I FAS in humans and animals [12]. Additionally, these parasite megasynthases appear to use acyl reductase domains to release the fatty acyl or polyketide chains as "long chain" alcohols, rather than using thioesterase (TE) to produce acids (Figure 1B). However, none of the apicomplexan genomes encodes any non-ribosomal peptide synthase (NRPS) that may act alone or together with PKSs to produce complex products.

Like most acyl reductase domains of the type I PKS and NRPS proteins, CpFAS1-R is located at the C-terminus and is defined by approximately 780 amino acids (aa) (Figure 1A). The first half of the domain contains conserved sequences and motifs characteristic to acyl reductase domains of PKSs and NRPSs, and to the fungal L-aminoadipate-semialdehyde dehydrogenase (AASDH), which include the Rossmann-fold NAD(P)H binding site "Gx_1-2_GxxG" and a reduction motif "GYxxSKWxxE" (Figure 1) [17-20]. However, the C-terminal half of the domain appears to be unique to the apicomplexans, as it is only homologous to the other apicomplexan FAS1 and PKS1 **R **domains, but not to any other species in the databases, nor does it contain any putative motifs.

Sequence comparisons indicated that the CpFAS1-R domain was more closely related to those of bacterial or fungal PKS/NRPS proteins. When CpFAS1-R domain was used as a query to search animal protein databases, there were no hits from vertebrate sequences. The only hits with significantly high identities are three invertebrate proteins annotated as oxidoreductase family or hypothetical proteins, i.e., *Ciona intestinalis*, (XP_002121624, E-value = 2.E-25), *Brugia malayi *(XP_001899674, 9.E-25), and *Trichoplax adhaerens *(XP_002112422, 8.E-15). Other invertebrate hits with much lower identities were a number of acyl-CoA reductases, mainly from insects such as *Drosophila mojavensis *(XP_002000602, 4.E-10) and *Culex quinquefasciatus *(XP_001847721, 1.E-9). This is in contrast to the hits from bacterial and fungal proteins, in which >420 and >170 hits displayed E-values at or smaller than 9.E-10 and 9.E-20, respectively.

**Figure 2:**
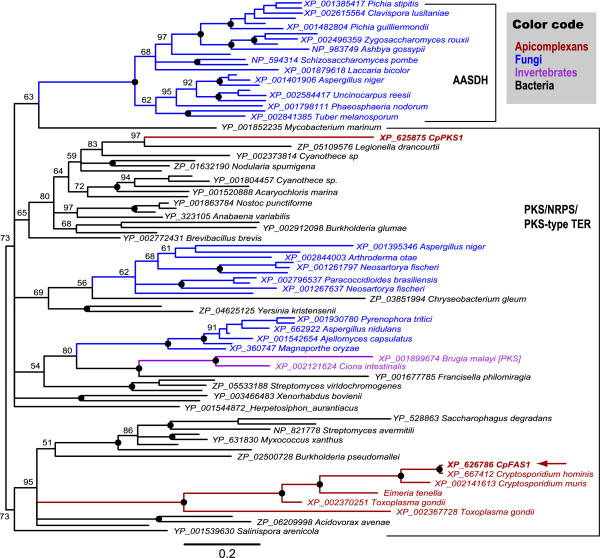
**Phylogenetic relationship of apicomplexan FAS/PKS reductase domains with orthologous sequences from bacterial and fungal PKS/NRPS, and fungal AASDH proteins as inferred by Bayesian inference (BI) method using a *WAG *+ *F***_**inv **_**+ *Γ*_(12) _model**. Detailed information on the model parameters and tree searches are described in the Materials and Methods. The position of CpFAS1-R is indicated by an arrow.

Phylogenetic reconstructions inferred from various bacterial and fungal reductases or **R **domains by Bayesian inference (BI) method clearly separated fungal AASDH from PKS/NRPS, in which major nodes were moderately to highly supported by the posterior probability (PP) values (Figure 2). Although fungal and bacterial PKS/NRPS formed several individual clusters, they were generally intermixed, possibly suggesting multiple origins of fungal PKS/NRPS genes. It is noticeable that putative oxidoreductases from two invertebrates (i.e., *Brugia malayi *and *Ciona intestinalis*) formed as a sister clade of γ-proteobacterial NRPS (*Francisella philomiragia*) (see branches colored in purple in Figure 2), suggesting a possible horizontal transfer of PKS/NRPS-like genes from prokaryotes to certain eukaryotes.

With the exception for CpPKS1-R, all apicomplexan FAS/PKS reductase domains (including CpFAS1-R) formed a single cluster that is fully supported by PP value (Figure 2, red branches). This group is then clustered together with several bacterial PKS/NRPS reductases. However, the CpPKS1-R domain alone was unexpectedly placed within another bacterial PKS/NRPS cluster. Although this observation is of some implication that CpFAS1 and CpPKS1 (or their R domains) might have different evolutionary origins, more rigorous analyses using additional domains are needed to truly make firm conclusions. Nonetheless, these observations support a bacterial affiliation of apicomplexan FAS/PKS **R **domains, as previously observed for the acyl transferase (AT) domains from CpFAS1 and CpPKS1 [12].

### CpFAS1-R domain's substrate preference

The **R **domain-catalyzed release of final products by bacterial and fungal PKS/NRPS polypeptides were mainly determined by analyzing the products in native or heterogeneous hosts expressing engineered PKS/NRPS genes [20]. There were several studies that directly observed the release/formation of products using recombinant PKS/NRPS modules [21-23]. However, there were no reported studies to directly assay the **R **domain activities and measure the kinetic parameters in vitro. On the other hand, it is impractical (if not impossible) to obtain sufficient pure *Cryptosporidium *materials for extensive biochemical analyses, and molecular tools are also unavailable to genetically manipulate this parasite. Therefore, heterogeneous expression systems and the use of recombinant proteins may be the only approaches currently available to study the biochemical feature of the CpFAS1 or CpPKS1 proteins and domains.

Suitable substrates are required to directly assay the **R **domain activity. However, there are some technical difficulties in preparing the native substrates for CpFAS1-R (i.e., very long chain fatty acyl-ACP). On the other hand, we have previously observed that the N-terminal AL domains from CpFAS1 and CpPKS1 could utilize LCFAs and CoA as substrates to form fatty acyl-CoA [13,24], and additionally, the CpFAS1-R domain is related to the acyl-CoA reductases (Figure 2). This prompted us to test whether fatty acyl-CoAs could also be used by CpFAS1-R as substrates to assay its activity.

**Figure 3:**
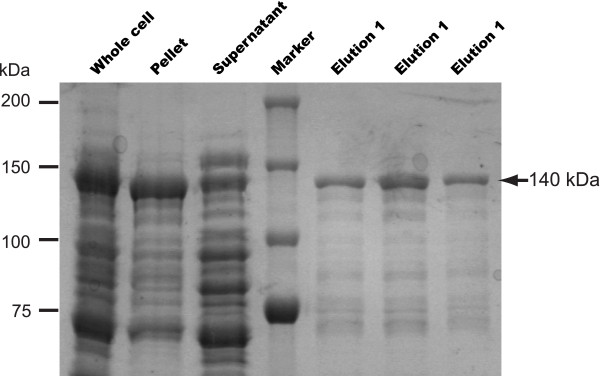
**SDS-PAGE analysis of recombinant CpFAS1-R domain as bacterial expressed maltose binding protein (MBP)-fusion protein before and after amylose resin-based chromatography purification as indicated on top of the gel**. The sizes of protein molecular markers and the MBP-CpFAS1-R protein are indicated. In a typical batch of purification, >90% of the proteins were the full-length CpFAS1-R.

**Figure 4:**
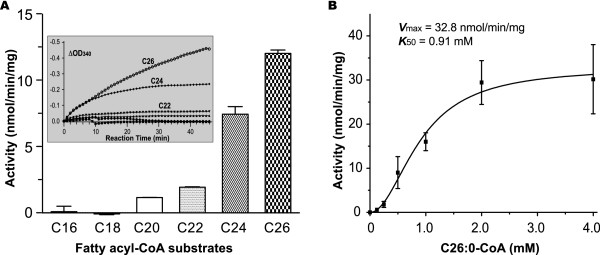
**Activity of recombinant CpFAS1-R as assayed using long to very long chain fatty acyl-CoA thioesters as substrates and NADPH as cofactor**. **A**) Substrate preference of CpFAS-R as displayed by activity. Inset shows the OD_340 _changes during the time course of reactions. **B**) Allosteric kinetic data of CpFAS1-R towards C26:0 hexacosanoyl CoA.

**Figure 5:**
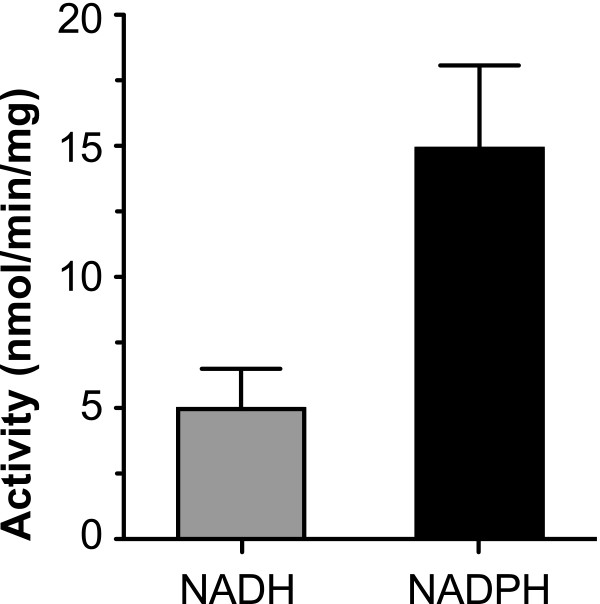
**Comparison on the activity of CpFAS1-R on NADPH and NADH using C26:0 hexacosanoyl CoA as the substrate**.

In this study, the recombinant CpFAS1-R was expressed and isolated into homogeneity as a maltose-binding protein (MBP)-fusion protein as previously reported (Figure 3) [13]. However, our first attempts using C16:0 palmitoyl-CoA as substrate and NAD(P)H as electron donors failed to detect any activity. Considering that the final CpFAS1 product(s) could be at least 6-carbons longer than the MCFAs or LCFAs loaded by the AL domain (i.e., after elongation by the three internal elongation modules) [12,13], we extended our analysis to include a wide range of all commercially available long chain to very long chain fatty acyl-CoAs, and eventually observed activities. In fact, CpFAS-Red was active on acyl-CoAs with ≥20-carbon chains, but inactive on substrates with 18 or fewer carbons (Figure 4A). The activities towards C20 and C22 acyl-CoAs were generally low, but well above the background, whereas activities towards C24 and C26 (the longest fatty acyl-CoA currently available) were much more apparent. Using NADPH as a cofactor, we have determined the kinetic parameters of CpFAS1-R towards C26-CoA that followed allosteric kinetics (Figure 4B). The Hill slope (***h***) was 2.0, indicating a positive cooperativity between the binding of cofactor and acyl-CoA. The maximum velocity (***V***_max_) and the substrate concentration to achieve 50% of the ***V***_max _value (***K***_50_) were determined to be 32.8 nmol min^-1 ^mg^-1 ^protein and 0.91 mM, respectively. The turnover rate (***K***_cat_) was ~4.6 min^-1^, indicating that CpFAS1-R could effectively transfer electrons from NADPH to this VLC fatty acyl-CoA, but at a low efficiency. CpFAS1-R could use both NADPH and NADH as cofactors, but it apparently prefers NADPH to NADH as co-assayed with C26-CoA (Figure 5).

## Discussion

As mentioned above, we have previously determined that the CpFAS1-AL domain prefers LCFAs as its substrates [13]. This may act as the first checkpoint to ensure correct fatty acids are loaded into this megasynthase for elongation. In this study, we have observed that CpFAS1-R domain could only utilize VLC fatty acyl-CoA, indicating that this **R **domain may act as a second checkpoint to ensure that correct lengths of products can be released.

The PKS/NRPS R domains may catalyze a single-step reduction (two-electron transfer) to produce fatty aldehyde, or a two-step reduction (four-electron transfer) to produce fatty alcohol [20]. Therefore, it is yet unclear whether CpFAS1-R-released products are VLC fatty aldehydes or fatty alcohols. We have repeatedly attempted, but failed to detect the CpFAS1-R-released acyl chains using thin layer chromatography (TLC) and mass spectrometry. On the other hand, TLC was able to distinguish C16 fatty alcohol and aldehyde using standards or preparations in controlled experiments (data not shown), or by other investigators [25]. It is possible that the limited amounts of VLC fatty acyl products were likely precipitated or aggregated after being separated from CoA to become insoluble for TLC or MS detection. However, since aldehydes are toxic to cells, and no additional VLC fatty acyl reductases are present in the *Cryptosporidium *genomes, we speculate that the final products produced by CpFAS1 are likely VLC fatty alcohols. Nonetheless, based on previously reported data and the present study, we are able to propose a general scheme of reactions catalyzed by the various domains of CpFAS1 as shown in Figure 1B.

Since the giant CpFAS1 and CpPKS1 are structurally and functionally different from humans Type I FAS, these parasite megasynthases may serve as novel drug targets. CpFAS1 and CpPKS1 also utilize **R **domains to release final products, which differs from human FAS that uses a thioesterase (TE) to product fatty acids. This unique feature suggests that like several other enzymatic domains, the reductase domains in CpFAS1 and CpPKS1 may also be explored as drug targets. The production of recombinant CpFAS1-R protein together with the novel assay developed in this study make it possible for the screening of inhibitors for potential drug development.

## Methods

### Sequence analysis

The CpFAS1-R domain protein sequence was used as a query to repeatedly search the NCBI genome and protein databases with various BLAST algorithms. More than 150 hits with e-values < 10^-20 ^were retrieved, which included discrete enzymes or reductase domains of multi-functional proteins. Multiple alignments were performed using a unix-based MUSCLE program (version 3.6), and short and nearly identical sequences were removed from the dataset. The dataset was further refined by additional multiple alignments with MUSCLE [26,27], and sampling of representative taxa to produce a final dataset containing 91 taxa and 184 aa positions. Conserved domains and motifs were identified and visualized as sequence logos by bits with the height of symbols within the stack indicates the relative frequency of each amino of the positions using WebLogo 3 (http://weblogo.threeplusone.com/) [28].

Bayesian inference (BI)-based phylogenetic reconstructions were performed with a parallel version of MrBayes program (version 3.1.2; http://mrbayes.csit.fsu.edu/) [29]). A WAG amino acid substitution model was used in the BI analysis. Among-site rate heterogeneity considered the fraction of invariance (*F*_inv_) and a discrete 12-rate gamma distribution (i.e., *WAG *+ *F*_inv _+ *Γ*_(12)_). At least 10^6 ^generation of searches were performed with two independent runs, each containing four chains running simultaneously. The current trees were saved every 100 generations, and the posterior probability (PP) values were calculated after the first 25% trees were discarded. The final consensus tree was visualized using a FigTree program (version 1.3.1; http://tree.bio.ed.ac.uk/software/figtree/).

### Biochemical analysis

The cloning of CpFAS1-R domain in a pMAL-c2x vector and the expression and purification of maltose-binding protein (MBP)-based recombinant CpFAS1-R (MBP-CpFAS1-R) has been previously described. The fusion protein was purified into homogeneity and used in our newly developed biochemical assays. Fatty acyl-CoAs with various carbon chain lengths (i.e., C16:0 to C26:0) were purchased from Avanti Polar Lipids, Inc. and used to replace native substrates acyl-ACPs. A typical reaction was performed in a 250 μl Tris.HCl (10 mM, pH 7.2) buffer containing 50 μg MBP-CpFAS1-R, 200 μM NADPH or NADH, 200 μM specified fatty acyl-CoA and 1 mM EDTA. The consumption of NAD(P)H was determined spectrometrically by measuring the decrease of absorbance at 340 nm (OD_340_) with a Multiskan Spectrum Microplate Spectrophotometer (Thermo Scientific) for up to 30 min. The kinetic parameters were measured similarly with varied concentrations of C26:0 hexacosanoyl CoA (0 to 4 mM). MBP was used as control for background subtraction. Each assay was performed in triplicate and independently at least twice. Enzyme activities and kinetic parameters were calculated using a Prism software (version 5.0c for Mac OS X) (GraphPad Software, Inc.). Nonlinear fit used following enzyme allosteric kinetics model:

$$\text{Y}=V_{\text{max}}{\left[\text{S}\right]}^{h}/\left(K_{\text{5}0}{}^{h}+{\left[\text{S}\right]}^{h}\right)$$

where **Y **is the enzyme activity, [**S**] is the substrate concentration, ***K***_50 _is the concentration of **S **to achieve 50% ***V***_max _value (similar to ***K***_m_), and ***h ***is the Hill slope.

## Abbreviations

AASDH: L-aminoadipate-semialdehyde dehydrogenase; ACP: acyl-carrier protein; AL: acyl-ligase; AT: acyltransferase; BI: Bayesian inference; DH: dehydrase; ER: enoyl reductase; FAS: fatty acid synthase; KR: ketoacyl reductase; LCFA: long chain fatty acid; MBP: maltose-binding protein; MCFA: medium chain fatty acid; NRPS: non-ribosomal peptide synthase; TLC: thin-layer chromatography; VLCFA: very long chain fatty acid; ORF: open reading frame; PKS: polyketide synthase; PP: posterior probability; R: reductase domain; TE: thioesterase.

## Authors' contributions

GZ designed the concept and experiments of this study, performed data analysis and prepared the manuscript. XS and MC expressed the fusion protein and performed experiments for determination of enzyme activity and kinetic data. All authors have approved the final manuscript.
